# Chinese expert consensus on wound repair of venous leg ulcers (2025 edition)

**DOI:** 10.1016/j.mmr.2026.100058

**Published:** 2026-07-30

**Authors:** Pi-Hong Zhang, Xiao-Bing Fu, Yue-Sheng Huang, on behalf of Wound Repair Professional Committee of Chinese Medical Doctor Association

**Affiliations:** a Department of Burns and Wound Repair, National Clinical Research Center for Geriatric Diseases, Xiangya Hospital of Central South University, Changsha 410008, China; b Research Center for Wound Repair and Tissue Regeneration, Medical Innovation Research Department, the PLA General Hospital, Beijing 100853, China; c Institute of Wound Repair and Regeneration Medicine, Southern University of Science and Technology School of Medicine, and Department of Wound Repair, Southern University of Science and Technology Hospital, Shenzhen 518055, Guangdong, China

**Keywords:** Venous leg ulcers, Wound repair, Debridement, Skin grafting, Flap transplantation, Expert consensus

## Abstract

Venous leg ulcers (VLUs) result from a multifactorial interplay of etiological factors, posing significant challenges in clinical management. Effective treatment requires a comprehensive assessment to identify the underlying pathophysiological mechanisms, followed by the implementation of an individualized, multidisciplinary approach that integrates the expertise of vascular surgeons, wound care specialists, and other pertinent healthcare professionals. Although both domestic and international vascular surgery societies, as well as other relevant specialties, have issued consensus statements on the diagnosis and treatment of VLUs or clinical guidelines for managing chronic lower extremity venous diseases, there remains a notable gap in systematic guidance specifically focused on wound repair for VLUs. In particular, standardized protocols for surgical wound repair are either lacking or insufficiently detailed in these documents. With the establishment and advancement of wound repair as a specialized field in China, Chinese experts have accumulated substantial clinical experience in the diagnosis and management of VLU-related wound healing. To standardize the surgical repair of VLUs and improve both therapeutic outcomes and patients’ quality of life, the Wound Repair Professional Committee of the Chinese Medical Doctor Association convened a panel of multidisciplinary experts for extensive deliberations. Based on a synthesis of current international and domestic evidence and clinical practice, the committee reached consensus on evidence-based recommendations for key issues in VLU wound management, including debridement techniques, indications, timing, and approaches to wound repair for VLUs. This consensus is intended to serve as a practical reference for clinicians involved in the care of patients with VLUs.

## 1 Background

Venous leg ulcers (VLUs) represent a highly prevalent and severe chronic vascular condition, affecting approximately 1% of the general population and rising to 3% among individuals aged over 80 years [1], [2]. Although up to 93% of VLUs achieve complete healing within 12 months, the remaining 7% progress to refractory ulcers persisting for more than 5 years; moreover, real-world clinical healing rates are substantially lower than this benchmark. Furthermore, the recurrence rate within three months after wound closure reaches as high as 80% [1], [2]. Beyond delayed healing and frequent recurrence, patients commonly suffer from excessive wound exudate, malodor, persistent pain, and restricted mobility, all of which considerably impair daily functioning. Long-term management also necessitates frequent clinical visits. Taken together, the complex interplay of these factors imposes a considerable medical and socioeconomic burden while profoundly compromising patients’ quality of life [3], [4], [5].

The pathogenesis of VLUs centers on unrelieved or ambulatory venous hypertension, which most commonly arises from deep venous thrombosis and subsequent venous incompetence. This sustained venous hypertension triggers a cascade of pathological changes, including lipodermatosclerosis, leucocyte plugging of the capillaries, tissue hypoxia, and microvascular dysfunction, ultimately leading to skin necrosis and ulceration [4], [6]. The clinical management of these patients represents a substantial challenge in contemporary medical practice, requiring robust multidisciplinary integration and in-depth collaborative research across vascular surgery, dermatology, foot and ankle surgery, and wound care specialists [4], [7]. Currently, vascular surgery departments are increasingly adopting minimally invasive endovenous techniques, such as endovenous ablation and iliac vein stent placement, for the surgical management of various vascular pathologies [8]. Moreover, clinicians in the field have achieved a consensus on the selection of safe and effective surgical strategies for venous interventions [2], [9], [10], [11]. However, wound repair of VLUs remains notably underemphasized. At present, the treatment of these wounds primarily relies on conservative approaches, which are often associated with prolonged wound healing.

In recent years, in response to public health needs and broader social development strategies, China has advanced the establishment and development of specialized wound repair departments. As a result, Chinese experts in wound repair have accumulated extensive clinical experience in the diagnosis and management of VLUs [12], [13]. To systematically develop standardized surgical repair strategies for VLUs, improve therapeutic outcomes, and enhance patients’ quality of life, the Wound Repair Professional Committee of the Chinese Medical Doctor Association convened a multidisciplinary panel of experts for comprehensive deliberations. These discussions were informed by a thorough review of both international and domestic literature on VLUs and supported by substantial clinical experience in diagnosis and treatment. The committee has established a consensus on the standardized management of VLUs, with a particular focus on providing evidence-based guidance for key issues of wound repair, including debridement techniques, indications, timing, and approaches to wound repair for VLUs. This consensus aims to serve as a clinically practical and reliable reference for healthcare professionals involved in the care of patients with VLUs.

## 2 Methods

### 2.1 Preparatory work for consensus development

#### 2.1.1 Expert selection

This consensus was developed under the guidance of the Wound Repair Professional Committee of the Chinese Medical Doctor Association through a rigorous and systematic process. It integrates a comprehensive review of international clinical guidelines, principles of evidence-based medicine, and extensive clinical expertise, reflecting the culmination of in-depth multidisciplinary deliberations. According to the principles of authority and representativeness, 31 experts with rich experience in the treatment of VLUs were selected for consultation. The panel comprises nationally recognized leaders in burn surgery, plastic surgery, wound repair, vascular surgery, nursing, and health statistics, drawn from top-tier tertiary hospitals and national-level scientific research institutions across China.

#### 2.1.2 Consensus working group

The development of this consensus involved a dedicated expert working group, which maintained close collaboration with experts. Additionally, a secretariat composed of 4 young clinical practitioners was established to support the non-clinical work associated with consensus development. The writing team completed the consensus registration and drafted the consensus. This consensus was completed with the guidance and assistance of methodological experts.

#### 2.1.3 Conflict of interest statement

Key considerations include the source of funding for the consensus development, potential financial interests in related commercial entities, and consultancy or employment relationships. All participants were required to complete a conflict-of-interest declaration to ensure transparency and integrity in the consensus process.

### 2.2 Questionnaire design and main content

#### 2.2.1 Framework for identifying clinical issues

**Fig. 1:**
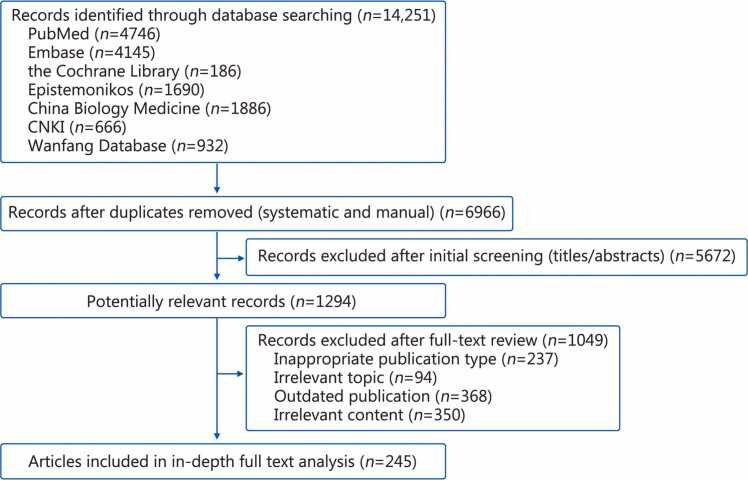
Literature screening results. CNKI. China national knowledge infrastructure.

A comprehensive and systematic literature search was conducted to collect relevant studies on etiological detection, clinical assessment, diagnosis, treatment, and preventing recurrence of VLUs, ensuring the comprehensiveness and authority of the included literature. The formulation of the search strategy was developed based on the core principles of the Preferred Reporting Items for Systematic Reviews and Meta-Analyses (PRISMA) guidelines. To cover both domestic and international research findings, the following databases were selected, PubMed, Embase, the Cochrane Library, and Epistemonikos (international databases), as well as China Biology Medicine, China National Knowledge Infrastructure (CNKI), and Wanfang Database (Chinese domestic databases), with the search period limited to May 31, 2025. Both medical subject headings (MeSH) terms and free words were used to construct the search strategy. MeSH terms included “venous ulcers” and “leg ulcer”; free words included “venous leg ulcers”, “VLUs”, “varicose ulcers”, and “venous stasis ulcers”. Boolean logical operators (AND, OR) were used to combine these search terms to optimize the search scope. The included literature types covered systematic reviews, randomized controlled trials (RCTs), observational studies (including cohort studies, case-control studies, cross-sectional studies, case series reports), and expert opinions. The actual search results are listed by Fig. 1. In addition, recent evidence published within the past year has also been added to the recommendation rationale.

Building upon the a forementioned systematic literature review, the working group further conducted small-scale expert interviews and targeted clinical surveys to identify and supplement critical clinically meaningful issues not addressed in available literature. The experts systematically collated and refined the focused clinical questions for VLUs, and developed an initial list of clinical issues in strict accordance with the population, intervention, comparison, and outcome (PICO) framework. Subsequently, the first round of the Delphi expert questionnaire was designed.

#### 2.2.2 Questionnaire content

The questionnaire consisted of 5 sections, which covered etiology and clinical assessment, differential diagnosis, treatment procedures and clinical pathways, as well as systemic and local therapies combined with wound repair strategies, including wound bed preparation, surgical debridement, skin grafting, and tissue flap transplantation. It was distributed to experts via an online platform.

The questionnaire collected experts’ demographic information, their recognition of each listed section, their familiarity with relevant issues, their judgment bases, and the degree of influence. To facilitate expert feedback for potential revisions, supplementary sections were included to capture expert suggestions. A 9-point scale was adopted for the section recognition scoring, with each indicator rated across 9 levels ranging from “extremely inappropriate” to “very appropriate”, and scores recorded sequentially as 1 to 9. Experts’ familiarity with each issue was classified into 6 levels, which (in descending order of familiarity) were 1.0, 0.8, 0.6, 0.4, 0.2, and 0 points. For the 4 judgment bases (theoretical analysis, practical experience, reference to domestic and foreign literature/data, and intuition), experts scored each issue based on three levels of reliance (i.e., high, moderate, and low) on each basis. The corresponding scores for these three reliance levels were 3, 2, and 1, respectively, while the weight coefficients of the 4 judgment bases were set as 0.2, 0.5, 0.2, and 0.1 in sequence.

### 2.3 Formation of recommendations

**Fig. 2:**
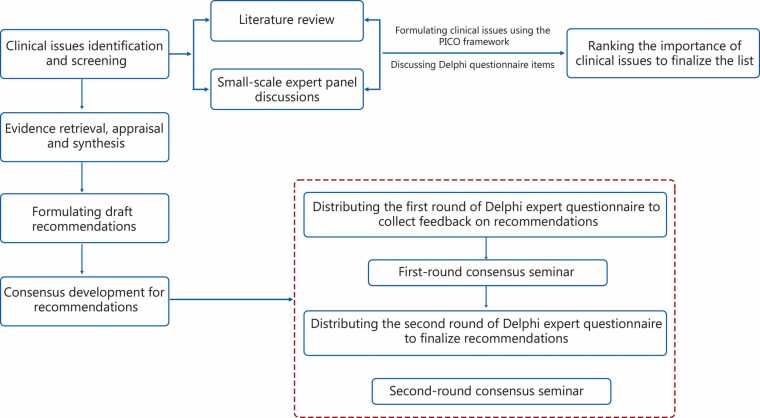
Results of the Delphi questionnaire survey. PICO. Population, intervention, comparison, and outcome.

Based on comprehensive literature reviews and their extensive clinical expertise, all participating experts completed two rounds of Delphi expert questionnaires (Fig. 2). Participants indicated their responses on a 5-point Likert-type scale with the following options: “recommended”, “somewhat recommended”, “neutral”, “somewhat not recommended”, and “not recommended”. They also had the option to provide supplementary written comments. The results of the first-round survey were compiled and sent back to participants for review in the second-round, and those completing both rounds were included in the final analysis. The Delphi process for each question concluded when agreement reached 70% or at the end of the second-round survey. For recommendations with 60%–69% agreement, we comprehensively documented the detailed dissenting rationales provided by the panelists (e.g., concerns about the applicability of the recommendation to VLU patients complicated by diabetes mellitus or peripheral arterial disease) and iteratively refined the recommendation wording in direct response prior to initiating a second-round survey [14], [15]. If the agreement rate still couldn’t reach 60% after the first survey or 70% after second-round survey, the relevant recommendations were excluded from the final manuscript. In terms of recommendation grading, Grade I was defined as reaching ≥90% panel agreement/recommend, while Grade II was assigned to items with 70%–89% agreement. Within Grade II, 70%–79% agreement was categorized as Grade IIb, and 80%–89% agreement as Grade IIa [16], [17], [18]. The results of the Delphi questionnaire survey are presented in **Additional File 1:** Table S1.

The levels of evidence (Table S2) were categorized in accordance with the European Society of Cardiology (ESC) grading system [2], [7]. The risk of bias and quality assessment of the included studies were conducted in strict accordance with internationally recognized and standardized evaluation criteria. For RCTs, the Revised Cochrane Risk of Bias Tool, Version 2 (RoB 2), was applied to ensure a systematic and transparent evaluation. For non-randomized studies of interventions, the risk of bias in non-randomized studies - of interventions (ROBINS-I) tool was utilized to maintain methodological rigor [19].

### 2.4 The process of consensus formulation

Launched in March 2024, this consensus initiative kicked off with a foundational meeting in Huangshan, Anhui Province, during which an expert committee composed predominantly of clinical specialists in burn and wound repair was established. At this meeting, the first draft of “Chinese expert consensus on wound repair of VLUs” was developed and subsequently registered on the International Practice Guidelines Registry Platform (Registration No. 2024CN1070). In November 2024, based on feedback collected from the first draft, the second draft of the consensus was presented at the Annual Meeting of the Wound Repair Professional Committee of the Chinese Medical Doctor Association held in Shenzhen, Guangdong Province. In April 2025, the final version of the consensus was discussed, reviewed, and formally approved through a voting process at a dedicated meeting held in Chizhou, Anhui Province. The manuscript was submitted for publication in August 2025.

The consensus development process adhered to the WHO’s 2014 Guideline Development Manual and the Chinese Medical Association’s 2022 manual for formulating clinical practice guidelines. This document is intended for domestic and international clinical practitioners involved in the management of patients with VLUs, including professionals from related specialties such as burn surgery, plastic surgery, vascular surgery, wound repair, and dermatology.

### 2.5 Statistical analysis

Statistical analyses were conducted using R version 4.4.1 (R Foundation for Statistical Computing, Vienna, Austria). The following indicators were computed: 1) the positive coefficient of the experts, defined as the questionnaire recovery rate; 2) the expert judgment coefficient (Ca) for each item, the familiarity coefficient (Cs), and the authority coefficient (Cr) [Cr = (Ca+Cs)/2]; 3) the mean score, standard deviation (SD), full score ratio (%), and coefficient of variation (CV, defined as the SD divided by the mean score) for each item. Kendall’s coordination coefficient was also calculated for each item to evaluate the consistency of expert scoring. A questionnaire survey of 31 target experts in the fields of burn and plastic surgery, wound repair, and vascular surgery was fully conducted, with a recovery rate of 100%, indicating a highly motivated level among the selected experts. The coefficients of variation for all the items were less than 0.15, indicating high consistency. Usually, Cr≥0.7 is defined as acceptable reliability. In this study, Cr was greater than or equal to 0.8 in all sections, indicating that this study has a high degree of expert authority (**Additional file 2:** Table S3).

## 3 Clinical issues and recommendations

### 3.1 Etiology and clinical assessment of VLUs

#### 3.1.1 Recommendation

The primary mechanism underlying the formation of VLUs is venous hypertension, which arises from lower limb venous reflux or obstruction. Although the clinical manifestations of VLUs are generally recognizable, wound healing often remains challenging. A comprehensive clinical assessment is essential for identifying the underlying etiology in patients with VLUs. Diagnostic tools such as venous Doppler ultrasound should be utilized, and classification systems including the Clinical-Etiology-Anatomic-Pathophysiologic (CEAP) classification and the Venous Clinical Severity Score (VCSS), can assist in evaluating the extent and severity of venous disease. **(Grade of recommendation: I; level of evidence: B)**

#### 3.1.2 Rationale

Accurate diagnosis of the underlying etiology of the disease is a prerequisite for standardized treatment. VLUs can be preliminarily diagnosed based on the location and morphology of the ulcer, as well as characteristic skin changes. In the early stage, VLUs typically present as irregular, superficial cutaneous erosions or ulcers in the ankle and/or lower calf, often accompanied by pale yellow exudate. The surrounding skin may exhibit pigmentation, stasis dermatitis, eczema, lipodermatosclerosis, soft tissue ossification, or atrophic blanche. These ulcers are frequently associated with varicose veins and tissue edema, with pain of varying intensity. Pain tends to worsen after prolonged standing and alleviates when the limb is elevated. VLUs are prone to complications such as infections (e.g., cellulitis and osteomyelitis) and are often difficult to heal. The duration of the ulcers can range from several weeks to several years, and in some cases, even decades [6].

In patients with VLUs, lower extremity venous pressure is elevated, and inflammatory cells and factors extravasate into the interstitial space, which in turn results in local stagnant hypoxia and impaired tissue nutrition. Matrix metalloproteinases (MMPs) can degrade the extracellular matrix, a process that not only contributes to ulcer formation but also hinders wound healing [20]. Prolonged chronic inflammation of the venous wall exacerbates valvular damage and degrades vascular elastic fibers, further causing venous dilation and increased reflux [21]. Given the complex pathogenesis of venous hypertension, encompassing multiple factors such as valvular dysfunction, outflow tract obstruction, arteriovenous malformations, and calf muscle pump (CMP) failure [22], a comprehensive functional assessment of the venous system is essential for patients with VLUs.

Currently, venous Doppler ultrasound is the most widely used non-invasive diagnostic tool. This examination can identify the anatomical location of venous lesions, such as in superficial veins, deep veins, or perforating veins, as well as assess the severity of the condition, and determine whether venous insufficiency is caused by reflux, obstruction, or a combination of both [23]. In certain cases, particularly when venous Doppler ultrasound fails to yield a definitive diagnosis, alternative imaging modalities may be utilized, including computed tomography venography (CTV), magnetic resonance venography (MRV), conventional venography (CV) (either antegrade or retrograde), and intravascular ultrasound (IVUS) [23], [24]. Additionally, tourniquet-assisted calf venous plethysmography can help differentiate between deep and superficial venous insufficiency or confirm the presence of isolated superficial venous incompetence [11].

The internationally recognized CEAP classification is applicable for assessing the severity of VLUs [25]. This classification has been widely popularized and implemented in clinical practice; it is conducive to the clinical staging of skin lesions, identification of anatomical locations, and analysis of pathophysiological changes in the abnormal venous system and further facilitates the formulation of subsequent targeted management decisions for VLUs at the root cause [25]. Moreover, the VCSS is widely utilized for the dynamic assessment of venous disease severity. The assessment primarily includes the following parameters, frequency of pain or discomfort episodes and their impact on daily activities; whether varicose veins or edema extend beyond the ankle or knee; whether skin pigmentation, inflammation, or induration involve areas beyond the ankle or the lower third of the leg; the number of active ulcers; ulcer duration (whether exceeding 3 months or 1 year); ulcer size (whether reaching 2 cm or 6 cm); and the use of pressure therapy. Each of these items is assigned a score ranging from 1 to 3 points [9].

### 3.2 Differentiation from arterial/mixed ulcers, cancerous ulcers, and ulcers caused by other etiologies

#### 3.2.1 Recommendation

VLUs may coexist with other comorbidities, such as arterial diseases and peripheral neuropathy. Differential diagnosis is essential to distinguish VLUs from ulcers of other etiologies, including those caused by arterial insufficiency, mixed etiologies, diabetes mellitus, lymphedema, trauma or infection, or autoimmune disorders. Furthermore, VLUs may undergo malignant transformation; therefore, biopsy should be performed when clinically indicated to confirm the diagnosis definitively. **(Grade of recommendation: IIa; level of evidence: C)**

#### 3.2.2 Rationale

In patients with VLUs, the posterior tibial and dorsalis pedis artery pulses are typically palpable. However, if these arterial pulses are diminished or non-palpable, concomitant arterial insufficiency should be suspected. In such cases, further evaluation using ankle-brachial index (ABI) measurement is indicated. An ABI value 0.9 indicates impaired arterial perfusion and raises suspicion for mixed-etiology ulcers [10]. Additionally, patients with severe VLUs may present with signs of peripheral neuropathy, including delayed limb movement, reduced sensitivity to temperature, and diminished light touch and vibration sensation. These neurological manifestations are correlated with a decrease in the total number of epidermal nerve fibers [26].

Patients with VLUs may have comorbid conditions, such as diabetes mellitus, lymphedema, and rheumatoid arthritis. In such cases, further diagnostic evaluations are necessary to confirm a definitive diagnosis [27].

Although biopsy can compromise blood flow and lead to impaired healing at the biopsy site, skin biopsy is important in nonhealing VLUs, serving roles including diagnosing cancer, staging the disease, identifying infectious processes, and assessing surrounding blood vessels [28]. Vascular histopathological results and duplex Doppler findings are used to identify segmental defects and potential surgical candidates. Both squamous cell carcinoma and basal cell carcinoma may develop into an ulcer in chronic venous stasis areas, potentially mimicking venous ulcers or complicating clinical course [4], [29]. Guidelines suggest performing a wound biopsy and histopathological examination only when there are atypical macroscopic features or lack of response to 4–6 weeks or longer of standard wound therapy [30].

### 3.3 Treatment procedures and clinical pathways of VLUs

#### 3.3.1 Recommendation

The management of VLUs requires a multidisciplinary strategy that addresses both clinical manifestations and underlying etiological factors. Timely surgical interventions, such as venous ablation, can correct venous reflux, promote wound healing, and reduce the risk of recurrence. Venous procedures should be promptly considered, either concomitantly with or following wound repair surgery. Additionally, adequate control of dermatitis and eczema is essential for optimizing outcomes of wound healing. **(Grade of recommendation: I; level of evidence: A)**

#### 3.3.2 Rationale

Although current conservative treatment strategies, such as active lower limb compression and regular wound dressing changes, can achieve wound healing in 50%–60% of VLU patients, challenges arise in managing those who are unresponsive to conservative therapy or have recurrent ulcers. These patients often face a range of clinical uncertainties. Treatment options may include venous surgery, the use of wound dressings containing bioactive components, local or systemic pharmacological therapies, and surgical wound repair. Furthermore, effective management requires collaboration across multiple disciplines, including vascular surgery, wound repair, foot and ankle surgery, dermatology, and wound therapy [8], [10], [31]. Therefore, an integrated, multidisciplinary approach is essential for the effective management of VLUs. This strategy should transcend traditional disciplinary boundaries and single-modal interventions, aiming to implement a comprehensive and systematic model of care. Continuous optimization of clinical pathways and full-course patient management is necessary to achieve the harmonious integration of “individualized treatment based on standardized protocols” and “standardized care tailored to individual needs” [32].

Research has indicated that timely implementation of venous surgical treatment, specifically, targeting the closure of incompetent varicose veins and perforating veins in the lower extremities, as well as ligation of perforating and superficial veins surrounding the ulcer, can effectively target venous hypertension at its source, enhance venous return, interrupt the pathophysiological progression of VLUs, prevent ulcer expansion, promote wound healing, and reduce recurrence rates [33]. Although established guidelines and expert consensus exist for the surgical management of venous malformations, it is crucial to emphasize that while vascular surgeons address venous abnormalities, concomitant wound care should also be prioritized [2], [9]. For patients with extensive VLUs that are unlikely to heal spontaneously, timely consultation with a wound repair specialist is recommended to assist in diagnosis and treatment. Similarly, patients whose ulcers fail to heal within a reasonable time following surgery should be promptly referred to wound repair professionals for further management. Particular emphasis should be given to the fact that when treating VLUs, doctors in the wound repair department should not limit their focus solely to the local ulcer area. Instead, they should adopt a holistic approach by investigating the underlying etiologies and conducting comprehensive diagnosis and treatment [7]. Collaboration with vascular surgeons is essential for conducting etiological assessments for potential venous disorders, followed by efficacious minimally invasive interventions, such as high ligation and stripping (HLS), endovenous laser ablation (EVLA), radiofrequency ablation (RFA), subfascial endoscopic perforating vein surgery (SEPS), venous VA reconstruction and sclerotherapy [8], [34], [35]. Endovenous procedures and stent implantation may also be indicated for post thrombotic syndrome (PTS) [36]. These interventions represent core strategies for achieving ulcer healing and preventing recurrence. Additionally, if VLUs are complicated by severe dermatitis or eczema, consultation with dermatologists should be arranged to guide the appropriate use of topical medications for symptom relief [37].

### 3.4 Local compression therapy for VLUs and its limitations

#### 3.4.1 Recommendation

Local compression therapy is beneficial for VLU healing and is considered a standard nursing intervention. This therapy involves selecting an appropriate wound dressing to maintain a moist wound environment and promote the healing process. However, its clinical application may be limited by specific patient-related factors. **(Grade of recommendation: IIa; level of evidence: A)**

#### 3.4.2 Rationale

Compression therapy serves as a fundamental intervention for VLUs and functions by improving venous hemodynamics through the application of controlled external pressure to the lower extremities [33], [38]. The standard procedure involves applying a pressure of approximately 35–40 mmHg at the ankle using elastic bandages or compression stockings, with a gradual pressure reduction to 17–20 mmHg near the knee. Patients are encouraged to maintain ambulation during compression therapy. After ulcer healing, the continued use of compression stockings with 30–35 mmHg of pressure is essential for preventing recurrence [2], [7], [9], [10], [39]. Although both endovenous surgery and local compression therapy achieve comparable ulcer healing rates, surgical intervention tends to yield lower recurrence rates [9]. Owing to the complexity of application, such as the need for up to 4 layers of elastic bandages, patient compliance with compression therapy using elastic bandages is often suboptimal. Consequently, compression stockings are frequently regarded as more cost-effective, effective, and practical alternatives [40]. In some instances, intermittent pneumatic compression (IPC) systems may also be utilized as an adjunctive intervention to alleviate symptoms through compression therapy [41].

Compression therapy has several limitations, including potential patient discomfort, excessive wound exudation, and technical challenges in proper application. It is contraindicated in patients with severe peripheral arterial disease [42]. When the therapeutic response to compression therapy is suboptimal, surgical intervention should be considered as an alternative treatment. Moreover, the use of appropriate wound dressings prior to pressure application can help maintain a moist wound environment, promoting more effective ulcer healing.

### 3.5 Systemic pharmacological adjunctive therapy for VLUs

#### 3.5.1 Recommendation

Pentoxifylline, flavonoids, and statins may be utilized as part of the systemic management strategy of VLUs. In the absence of infectious complications, antibacterial agents, whether administered locally or systemically, are generally not indicated for VLU management. When differentiation between bacterial colonization and true invasive infection is needed and when identification of potential pathogenic microorganisms is necessary to guide antimicrobial therapy, tissue specimens may be collected for semiquantitative or quantitative bacterial cultures. **(Grade of recommendation: IIa; level of evidence: C)**

#### 3.5.2 Rationale

Many systemic pharmacological agents, including pentoxifylline, flavonoids, statins, aspirin, thromboxane A2 antagonists, prostaglandins, and prostacyclin analogs, have been investigated as adjunctive therapies to promote VLU healing. While the therapeutic efficacy of pentoxifylline (400 mg three times daily) is superior to that of placebo in promoting ulcer healing (*RR* 1.70, 95% CI 1.30–2.24) [43], several other drug classes also exhibit modest therapeutic benefits in VLU management. Diosmin, a flavonoid compound administered at a dosage of 1 g per day, has been shown to alleviate symptoms and reduce edema associated with chronic venous insufficiency [44]. Statins may facilitate ulcer healing and improve patients’ quality of life [45]. Sulodexide enhances local microcirculatory exchange through its antiplatelet, anti-inflammatory, antiproteolytic and protective effects on the glycocalyx endothelial layer, thereby promoting the healing of VLUs [46]. In contrast, oral aspirin has not demonstrated clinically meaningful efficacy in ulcer management and is therefore not recommended as a routine therapeutic agent.

Unless VLUs are complicated by invasive or systemic infections, the routine administration of antibacterial agents, whether for local or systemic treatment, is not recommended [28]. When VLUs are complicated by invasive infections such as erysipelas, cellulitis, superficial phlebitis, or lymphangitis, clinical features may include persistent local pain, a dull appearance of the wound bed, increased exudate production, delayed wound healing, and even wound expansion, abscess formation, and malodor. Systemic manifestations such as malaise and loss of appetite may also occur. Wound swab bacteriological testing often fails to differentiate between bacterial colonization and deep tissue infection. If these severe invasive infections occur, a wound bed biopsy specimen should be collected following thorough wound cleansing with normal saline for bacterial culture. This procedure enables precise identification of pathogenic microorganisms and provides an evidence-based basis for the selection of appropriate antibacterial agents [47].

### 3.6 Wound bed preparation prior to VLU wound repair

#### 3.6.1 Recommendation

VLUs typically exhibit a pronounced edematous appearance. Prior to surgical interventions such as skin grafting, comprehensive and adequate wound bed preparation is essential. This multimodal therapeutic strategy includes wound cleansing, debridement of necrotic tissue, appropriate dressing selection and application, effective management of dermatitis and eczema, rational use of growth factors and platelet-rich plasma (PRP), topical administration of traditional Chinese medicine (TCM) preparations, and the application of negative pressure wound therapy (NPWT), along with other evidence-based interventions. **(Grade of recommendation: IIa; level of evidence: C)**

#### 3.6.2 Rationale

Wound bed preparation plays a vital role in promoting effective VLU repair. Initially, wound cleansing should be performed using non-toxic agents that do not compromise viable tissue. Normal saline is the most utilized solution for this purpose. In cases of mild local inflammation, characterized by redness, swelling, and increased exudate, when infection is suspected, antimicrobial agents such as iodophor, povidone-iodine, and silver-containing antimicrobial preparations may be utilized [48], [49], [50]. Polyhexamethylene biguanidine hydrochloride (PHMB) is another suitable option for wound cleansing because of its broad-spectrum antibacterial properties and low risk of inducing bacterial resistance; however, it is associated with high costs [48]. Although widely used, chlorhexidine is not recommended for chronic ulcers because of its potential cytotoxicity, which may impair collagen synthesis and inhibit tissue regeneration [51]. When a thin layer of necrotic tissue persists on the wound surface, topical enzymatic debriding agents or TCM preparations with eschar-removal properties can be applied to facilitate the dissolution and clearance of necrotic tissue [52]. In recent years, TCM external preparations have shown considerable promise in the management of VLUs, owing to their favorable attributes, including ease of application, proven therapeutic efficacy, cost-effectiveness, and a variety of formulation options. A range of dosage forms, such as lotions, ointments, powders, and sprays, along with representative agents including Moisture Exposed Burn Ointment, Hong You Ointment, Sheng Ji Powder, and Zihua Burn Ointment, have been developed and are increasingly accepted in clinical practice [53]. Integrated traditional Chinese and Western medicine approaches may enhance wound healing through multi-target mechanisms, such as improvement of lower limb vascular function, promotion of venous return, acceleration of hemostasis, reduction of inflammatory responses, pain alleviation, and stimulation of tissue proliferation and regeneration [54], [55].

Furthermore, the appropriate selection and clinical application of advanced wound dressings can accelerate wound bed preparation in VLUs and enhance the healing process. Hydrogel and hydrocolloid dressings promote effective autolytic debridement; alginate dressings are particularly effective for absorbing wound exudate; polyurethane foam dressings provide protection for granulation tissue; and polyurethane film or collagen-based dressings facilitate epithelialization at the wound margins [56]. In clinical practice, dressing selection should be guided by multiple clinical factors, including wound location, size, depth, exudate volume, odor, presence of infection or allergic reactions, patient comfort, ease of dressing changes and frequency, and cost considerations [31], [50], [57]. These dressings may also be utilized in combination with compression therapy to further promote ulcer healing. Despite their potential benefits, the Cochrane review revealed no significant advantage of any specific dressing type over low-adherence dressings when both were used in combination with compression therapy [31].

In addition, the application of certain growth factors, such as epidermal growth factor (EGF), fibroblast growth factor (FGF), and granulocyte-macrophage colony-stimulating factor (GM-CSF), as well as PRP can promote granulation tissue formation in VLUs, thereby accelerating the VLU wound healing process [58], [59], [60], [61], [62], [63], [64], [65], [66]. Research has demonstrated that a plasma-derived product containing 6 growth factors involved in tissue regeneration may also contribute to improved VLU healing outcomes [67].

NPWT can serve as an adjunctive therapy in wound bed preparation, particularly for refractory VLUs [68], [69], [70]. When utilized in wound management, NPWT primarily delivers sub-atmospheric pressure, typically ranging from −50 to −125 mmHg, to the wound site, thereby eliciting multiple beneficial effects, including microstrain, macrostrain, edema control, effective drainage management, decreased bacterial burden, granulation tissue formation, promotion of neoangiogenesis, and wound stabilization [68], [70]. Furthermore, the cyclic alternating negative pressure mode has been shown to be significantly more effective than the single negative pressure mode in improving peri-wound TcPO₂, lowering wound pH, reducing exudate volume, and alleviating pain [41]. As an optimized variant of traditional NPWT, negative pressure wound therapy with instillation and dwell time (NPWTi-d) has been successfully utilized for rapid wound bed preparation in patients with VLUs [71], [72], [73], [74]. During treatment, normal saline or 0.125% hypochlorite solution (Dakin’s solution) was instilled periodically with a solution dwell time of 10 min, followed by NPWT cycles of 2–2.5 h at −100 to −125 mmHg, and dressings were changed every 72 h. This combined modality effectively controls heavy exudation, reduces bacterial burden, and debrides devitalized tissues, and may be used either as an adjunct or an alternative to surgical debridement.

### 3.7 Surgical debridement of VLUs

#### 3.7.1 Recommendation

For VLUs with extensive necrotic tissue, timely surgical debridement is recommended. Common techniques include radical debridement, conservative debridement, ultrasonic debridement, hydrodynamic debridement, and laser debridement. Radical debridement with a depth reaching the deep layer of the dense fibrous plate and extending beyond the liposclerotic region is more beneficial for promoting wound healing and ensuring skin graft survival. This strategy deserves increased clinical attention and broader application. **(Grade of recommendation: IIa; level of evidence: C)**

#### 3.7.2 Rationale

Debridement is critical for reducing the burden of necrotic tissue and promoting the formation of healthy granulation tissue. Surgical debridement can transform chronic ulcer wounds to an acute wound state, thereby enabling more effective healing [72]. This procedure eliminates necrotic tissue, fibrin deposits, and foreign bodies, disrupts biofilm formation, improves the local biodistribution of antibacterial agents, and aids in preventing biofilm reformation [75], [76]. Surgical debridement should be performed under anesthesia and is indicated for larger VLUs with extensive devitalized and/or necrotic tissue involvement.

Surgical debridement for VLUs requires strict adherence to the principle of appropriate, tissue-sparing resection. While radically resecting pathological tissues that impede wound healing, utmost care must be exercised to avoid injuring critical anatomical structures such as nerves and tendons [77]. The depth and extent of debridement are of particular importance. On the one hand, venous ulcers are often complicated by dense deep tissue fibrosis, characterized by a significant increase in mature collagen content, which impedes wound healing. Therefore, debridement should extend past the dense fibrous plate. On the other hand, the skin surrounding chronic nonhealing venous ulcers frequently presents with subcutaneous liposclerosis, a pathological condition that adversely affects the wound healing process and thus should be removed [78], [79]. Following adequate debridement, coverage with split-thickness skin grafts (STSGs) is recommended [79], [80].

Among other surgical debridement methods, conservative surgical debridement is commonly utilized. Under local anesthesia, instruments including scalpels, scissors, and curettes are employed to resect devitalized tissue from the wound surface and its adjacent area [81]. Ultrasonic debridement utilizes the cavitation effect to induce the rupture, fragmentation, and emulsification of necrotic tissue [82]. Hydrodynamic debridement utilizes the Venturi effect generated by high-speed water jets to remove devitalized tissue from the wound surface. Laser debridement can vaporize necrotic tissue down to the healthy tissue plane and create microperforations in the wound bed to promote granulation tissue formation and expedite healing [83], [84], [85]. For certain VLUs that are not amenable to immediate skin grafting following surgical debridement, NPWT can be applied to promote granulation tissue formation, subsequently enabling split-thickness skin grafting [86].

### 3.8 Indications for skin grafting in VLUs

#### 3.8.1 Recommendation

For patients with VLUs presenting with a large ulcer area, a prolonged disease course, or suboptimal response to conservative treatment, wound bed preparation should be promptly initiated in combination with vascular intervention, followed by timely skin grafting to promote wound repair. **(Grade of recommendation: IIa; level of evidence: C)**

#### 3.8.2 Rationale

Although a Cochrane review demonstrated that there is limited high-quality evidence supporting autologous skin grafting for VLUs [87], this procedure remains widely employed in clinical practice for the management of many VLUs. When VLUs is unresponsive to conservative management strategies, including compression therapy, wound cleansing, debridement, and appropriate dressing use, or show no improvement over a 4-week period, autologous skin grafting may act as a feasible and effective therapeutic option [84], [85], [86], [87], [88]. Generally, larger ulcers with an area exceeding 25 cm² are considered appropriate candidates for skin grafting [89].

Amann-Vesti *et al.* [90] have suggested that to address the prolonged postoperative healing period frequently associated with relatively large ulcers (which may last for several months), patients with ulcer diameters exceeding 3 cm should undergo local wound dressing changes prior to surgical intervention. Once the granulation tissue on the wound surface is viable, autologous free skin grafting can be performed simultaneously with venous lesion procedures such as SEPS. This combined approach may promote complete ulcer healing within approximately three weeks [90]. In fact, while large ulcer size, deep wound involvement, and prolonged disease course are commonly recognized as indications for skin grafting in VLUs, the key determinants of successful graft survival include adequate wound bed preparation, vigorous granulation tissue formation, thorough radical debridement, and sufficient wound bed vascularity [73], [80].

Additionally, NPWT has been increasingly utilized in the fixation of STSGs [91]. In skin grafting procedures, NPWT enables uniform distribution of negative pressure across the wound surface, facilitating consistent adherence of the graft to the recipient site. This mitigates adverse effects on neovascularization induced by excessive shear forces between the graft and the wound bed, thereby increasing graft survival rates [91], [92]. In a clinical study involving 18 patients with VLUs who underwent NPWT-assisted skin grafting, the mean skin graft survival rate reached 92% (range, 80%–100%) after removal of the negative pressure device, with no infections observed at the graft sites [91].

### 3.9 Optimal timing for surgical intervention for VLUs

#### 3.9.1 Recommendation

The optimal timing of surgical intervention for VLUs and the sequence of this repair relative to the treatment of pathological veins should be determined based on the condition of the affected blood vessels, selected treatment modalities, wound location and size, and presence of infection. Whenever feasible, debridement and skin grafting for VLUs should be performed concurrently with minimally invasive procedures targeting underlying venous pathological changes. **(Grade of recommendation: IIa; level of evidence: C)**

#### 3.9.2 Rationale

Following the active management of superficial venous axial reflux, pathological perforating veins, and deep venous disorders, most ulcers can achieve spontaneous healing [4], [33], [93]. However, this process is often time-consuming, and prolonged wound care imposes a substantial burden on healthcare resources [4], [33]. For certain wounds that remain unhealed despite prolonged conservative treatment, surgical interventions such as skin grafting are recommended to promote wound repair [80]. In some cases, when large ulcers are accompanied by superficial venous disease, wound bed preparation and wound repair should be prioritized prior to addressing the underlying venous abnormalities to mitigate the risk of infection [21], [94]. The sequence of therapeutic strategy implementation may vary across clinical settings, with the timing of surgical wound repair guided primarily by clinical judgment and individual patient factors [7]. In recent years, for patients with lower extremity varicose veins complicated by VLUs, combined strategies involving minimally invasive vascular interventions, such as EVLA [33], [95], in conjunction with split-thickness skin grafting have yielded favorable outcomes, supporting their broader adoption in clinical practice.

### 3.10 Wound repair strategy for VLUs

#### 3.10.1 Recommendation

Techniques such as the application of STSGs, which can also be processed into meshed or microskin grafts, as well as the adoption of autologous epidermis and bilayer artificial skin grafts are effective therapeutic options for VLUs repair. When clinically indicated, the incorporation of NPWT can promote wound healing in patients with VLUs. However, this skin-graft approach necessitates thorough surgical debridement of the wound area and meticulous wound bed preparation. The primary objective is to remove the underlying fibrotic plate and ensure the absence of visible liposclerotic tissue surrounding the wound, thereby promoting optimal survival and integration of the grafted skin. **(Grade of recommendation: IIa; level of evidence: C)**

#### 3.10.2 Rationale

A study has shown that in the management of VLUs, skin grafting achieves a higher success rate than other chronic leg ulcer types [80], indicating that autologous split-thickness skin grafting is an efficacious therapeutic approach for wound repair in VLU patients. In certain cases, meshed skin grafts may be applied, or medium-thickness skin grafts can be harvested from the inguinal region during high ligation of the great saphenous vein for transplantation [96]. Autologous epidermal grafting is also utilized for VLU repair and confers several advantages over conventional skin grafting techniques, including no requirement for anesthesia, procedural simplicity, lower cost, and reduced failure rates [97]. Additionally, microskin grafts can rapidly induce wound re-epithelialization, facilitate uneventful wound healing, and cover relatively large ulcer areas with only a small donor skin sample [98].

The survival of grafted skin is primarily determined by the adequacy of local surgical debridement and the thoroughness of wound bed preparation [80], [81]. Emphasis should be placed on the complete removal of the dense fibrotic plate and the excision of tissue outside the liposclerotic zone to achieve effective radical debridement [99]. This approach not only markedly improves the survival rate of skin grafts but also significantly reduces the recurrence rate of ulcers. A growing body of literature supports the view that the combination of free skin grafting and NPWT is more beneficial for both graft survival and overall wound healing [100], [101], [102]. In clinical practice, an increasing number of VLU patients are undergoing NPWT as an adjunctive treatment subsequent to skin grafting.

In addition, researchers performed a retrospective analysis of 17 clinical studies involving a total of 1034 patients. The findings showed that, compared with simple dressings, only bilayer artificial skin grafts exhibited significantly better efficacy in promoting venous ulcer healing. The clinical advantages of other skin graft types, such as autologous skin grafts and cryopreserved or fresh allogeneic grafts, still warrant further investigation and validation [103]. Stone *et al*. [104] demonstrated that a bioengineered bilayer living cell construct induces VLU healing by orchestrating the transition of VLUs from a chronic nonhealing ulcer microenvironment to a unique healing environment analogous to acute healing wounds, including the regulation of inflammation and growth factor signaling, the activation of keratinocytes, and the attenuation of Wnt/β-catenin signaling; notably, this skin substitute has been approved by the US Food and Drug Administration for the treatment of VLUs.

Several cell/tissue-based products have also been applied in the treatment of refractory VLUs, including allografts, animal-derived extracellular matrix products, human-derived cellular products, and human amniotic membrane derivatives [105], [106]. Moreover, treatments such as autologous keratinocyte-fibrin suspension transplantation have shown preliminary therapeutic potential for VLUs [107], [108]. Research has confirmed that extracellular vesicles from stem cells or autologous plasma rich in growth factors such as transforming growth factor-β1 can promote angiogenesis, granulation growth, and wound healing in VLUs [82], [83]. However, owing to multiple constraints including sophisticated manufacturing processes, considerable economic costs, and potential safety concerns, the favorable efficacy of these advanced cellular and biological interventions has thus far only been demonstrated in preclinical studies and early-phase clinical trials [105], [106], [107], [108]. Further investigations are therefore warranted to validate their economic viability, resource accessibility, and cost-effectiveness profile prior to their widespread clinical translation.

In summary, different shapes and sizes of autografts, allografts and biosynthetic scaffolds are beneficial for the healing of chronic or refractory VLUs. These transplant materials can be selected based on the size, shape, and base condition of the wound, as well as the patient’s general condition and cost-effectiveness.

### 3.11 Role of tissue flap transplantation in VLUs in enhancing venous return and reducing the risk of ulcer recurrence

#### 3.11.1 Recommendation

Although the effect of tissue flap transplantation on venous hemodynamics in VLU patients remains a topic of debate, the application of various tissue flap techniques, including skin flaps, myocutaneous flaps, and greater omental flaps, has exhibited superior efficacy in repairing complex VLUs presenting with exposed bones, joints, or tendons, as well as recurrent and refractory conditions. **(Grade of recommendation: IIb; level of evidence: B)**

#### 3.11.2 Rationale

A wide array of flap coverage techniques is available for defects of the lower extremities, including cross-leg flaps, local cutaneous or fasciocutaneous flaps based either proximally or distally, distally based muscle flaps, and free flaps [109], [110], [111], [112]. Among these options, free flaps enable replacing the irreversibly scarred, poorly vascularized tissue bed with healthy tissue containing multiple competent microvenous valves and a normal tissue microcirculation, thus more effectively addressing the hemodynamic and tissue-related pathophysiological factors underlying refractory VLUs [109], [110]. As early as 1997, Weinzweig *et al*. [109] described the use of free myocutaneous flaps to reconstruct wounds subsequent to extensive resection of refractory VLUs and the surrounding liposclerotic tissue. This study involved 20 myocutaneous flap procedures performed on 19 wounds in 18 patients, with a flap survival rate of 90%. The average follow-up period was 32.7 months (ranging from 8 to 65 months), during which time only one patient developed ulcer recurrence adjacent to the flap [109]. The most commonly utilized free flaps include the rectus abdominis flap and the latissimus dorsi myocutaneous flap, followed sequentially by the gracilis and serratus anterior muscles. In general, the latissimus dorsi flap was selected for coverage of large-sized defects, the rectus abdominis or “split” latissimus dorsi flap for moderate-sized defects, and the serratus anterior and gracilis flaps for small-sized defects [109], [111]. Recently, Cavadas *et al*. [112] reported that following circumferential radical resection of severe VLUs in 5 cases, free greater omental transplantation combined with skin grafting was adopted for wound reconstruction. The results showed a 100% survival rate for the transplanted tissue flaps. The average follow-up duration was 8 years (ranging from 4 to 15 years), during which one patient developed an ulcer within the 2-year follow-up period and subsequently achieved healing via conservative treatment [112]. The greater omental flap is the widest soft tissue flap described (excluding fillet flaps) and can readily achieve circumferential coverage of 360° defects in the lower extremities.

The use of distally based sural flaps for VLUs repair has been reported [108]. All 12 patients were successfully treated, with no flap failures noted. The average follow-up duration was 19.7 months, and no instances of ulcer recurrence were recorded [110]. Additionally, the use of reverse superficial sural artery flaps or perforator-based propeller flaps for wound coverage in VLUs has yielded relatively satisfactory outcomes [113], [114]. The greatest advantage of a sural flap lies in its technical simplicity, enabling reliable execution even by surgeons without plastic surgery specialization, thus making it particularly valuable in resource-constrained medical institutions.

Based on these findings, it was hypothesized that such tissue flap transplantation may exert a positive effect on the venous hemodynamics of patients with VLUs. However, Song [111] conducted a retrospective analysis of 5 non-controlled cohort studies involving 56 patients with 62 refractory VLUs, on which 64 free-flap transplantations were performed. The average patient age was 50 years (range, 17–76 years), and the mean wound defect area was 153.3 cm² (range, 24–600 cm²). Among these procedures, there were 16 latissimus dorsi flaps, 16 rectus abdominis flaps, 30 free perforator flaps, and two greater omental flaps. The flap survival rate attained 95%. During the follow-up period of 24 to 125 months, no ulcer recurrence was noted within the flap area; however, 20 patients (35.7%) developed new ulcers outside the flap boundary [111].These data suggest that there is currently no evidence to support the view that flap transplantation improves venous hemodynamics in patients with VLUs, thereby preventing ulcer formation outside the reconstructed region [111].

### 3.12 Postoperative management of VLU repair and preventive strategies for ulcer recurrence

#### 3.12.1 Recommendation

Following VLU repair surgery, patients should be encouraged to enhance adherence to medical advice. They are advised to comply with compression therapy, leg elevation, short-distance ambulation, and progressive resistance exercises as part of a comprehensive postoperative regimen aimed at preventing ulcer recurrence. **(Grade of recommendation: I; level of evidence: A)**

#### 3.12.2 Rationale

In the microcirculation of the skin on the legs of patients with VLUs, anatomical changes in capillaries are commonly observed. Even after undergoing STSGs, these patients continue to exhibit significant microcirculatory abnormalities. These abnormalities are characterized by tissue hypoxia, impaired lymphatic regeneration, epidermal thickening, and dermal thinning [115]. Moreover, patients remain at high risk for ulcer recurrence, with reported recurrence rates ranging from 54% to 78%. This high recurrence rate imposes a substantial socioeconomic burden in terms of healthcare costs, occupational impact, and decreased quality of life [116]. Comprehensive preoperative etiological evaluation, appropriate venous surgical intervention, and long-term adherence to compression therapy are key strategies for preventing recurrence [6], [33], [93]. Elevating the legs above heart level (3–4 times daily, at least 30 min each), engaging in short-distance ambulation (3–4 times daily) to improve CMP function, and performing progressive resistance exercises have been shown to be beneficial [108]. Furthermore, multiple objective metrics confirm that the participants maintained consistent adherence to the exercise protocol, and the training was implemented as intended across all exercise domains [117].

Additionally, robust social support systems and high levels of patient adherence to medical advice have been shown to facilitate the prevention of VLU recurrence [118]; this includes sustained strengthening of health education via new media networks and offline health lectures, gradually improving patient health records and full-course patient management through online visits and outpatient follow-up visits, placing great emphasis on adherence to VLU compression therapy, implementing nurse-led community service projects to reinforce follow-up management of VLUs, and formulating feasible management plans for preventing VLU recurrence [119], [120].

## 4 Conclusions

The management of VLUs is a multidisciplinary endeavor that involves collaborative efforts across multiple medical specialties. It specifically encompasses interventions targeted at eliminating or alleviating venous hypertension, such as compression therapy and surgical correction of venous abnormalities; the administration of topical and systemic pharmacotherapeutic agents that promote wound healing; and local surgical interventions, such as debridement, skin grafting, and flap transplantation.

**Fig. 3:**
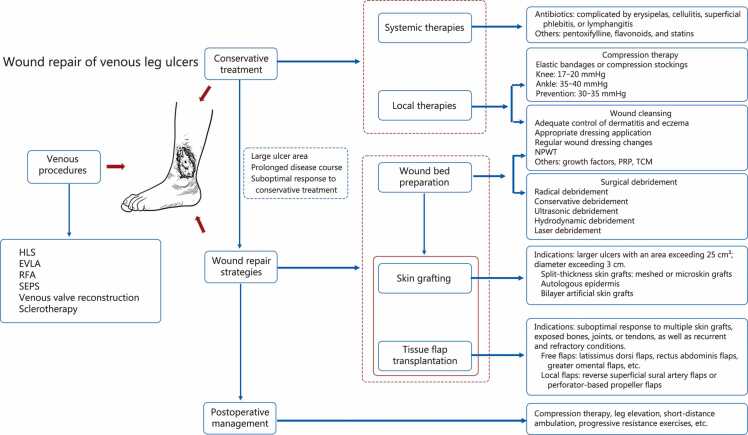
Wound repair of venous leg ulcers. HLS. High ligation and stripping; EVLA. Endovenous laser ablation; RFA. radiofrequency ablation; SEPS. Subfascial endoscopic perforating vein surgery; PRP. Platelet-rich plasma; TCM. Traditional Chinese medicine.

First, removing existing barriers to collaboration among different professional disciplines involved in the treatment of VLUs is essential. Enhancing interdisciplinary cooperation and refining patient-centered consultation and referral mechanisms are crucial steps toward achieving effective integrated care. Moreover, integrating human resources to establish a dedicated medical team or a specialized VLU treatment unit consisting of multidisciplinary professionals can be highly beneficial. This approach allows for the full utilization of multidisciplinary expertise; facilitates the implementation of personalized, optimized treatment regimens and sequential interventions; and ultimately promotes timely wound healing at a relatively low cost, while helping to prevent or minimize ulcer recurrence (Fig. 3).

Second, it is imperative to develop a research design template that is more efficacious in demonstrating therapeutic efficacy, establish a clinical trial network platform capable of sustaining long-term follow-up, and further promote high-quality clinical studies on the comparative effectiveness of treatment protocols. These initiatives will enable more scientifically rigorous observations and more accurate comparisons among homogeneous patient populations, thereby facilitating the identification of optimal treatment interventions [93].

Third, clinical and basic research on VLUs should be enhanced to achieve a deeper understanding of their underlying etiologies and pathophysiological mechanisms [121]. Moreover, innovative approaches and cutting-edge technologies from the fields of regenerative medicine and wound repair should be rationally integrated into VLU management strategies [122]. The aim is to enhance wound healing outcomes and offer more effective therapeutic options for patients with VLUs.

Finally, while this consensus focuses on the establishment of a standardized diagnosis and treatment system for VLUs, its core conclusions offer valuable implications for other prevalent ulcer-related fields. Although distinct types of ulcers exhibit substantial differences in their etiological mechanisms and lesion locations, they share common ground in terms of their pathological feature of chronic non-healing, core therapeutic goals centered on wound healing, functional preservation, and limb salvage, as well as the implementation rationale of multidisciplinary collaboration combined with long-term follow-up management. On this basis, it is feasible to establish logical connections in diagnosis and treatment among different ulcer types, thereby laying a theoretical and practical foundation for the further development of a universal diagnosis and treatment framework for chronic ulcers.

## Abbreviations

ABI: Ankle-brachial index

CEAP: Clinical-Etiology-Anatomic-Pathophysiologic

CMP: Calf muscle pump

CV: Conventional venography

CTV: Computed tomography venography

ESC: European Society of Cardiology

EGF: Epidermal growth factor

EVLA: Endovenous laser ablation

FGF: Fibroblast growth factor

GM-CSF: Granulocyte-macrophage colony-stimulating factor

HLS: High ligation and stripping

IPC: Intermittent pneumatic compression

IVUS: Intravascular ultrasound

MMPs: Matrix metalloproteinases

MRV: Magnetic resonance venography

NPWT: Negative pressure wound therapy

PHMB: Polyhexamethylene biguanidine hydrochloride

PRP: Platelet-rich plasma

PTS: Post-thrombotic syndrome

RFA: Radiofrequency ablation

SD: Standard deviation

SEPS: Subfascial endoscopic perforating vein surgery

SG: Skin graft

STSGs: Split-thickness skin grafts

TCM: Traditional Chinese medicine

VCSS: Venous clinical severity score

VLUs: Venous leg ulcers

WBP: Wound bed preparation

## Ethics approval and consent to participate

Not applicable.

## Authors’ contributions

PHZ, XBF and YSH as the main person in charge of the consensus, presided over the expert seminar, formulated the consensus agenda, and coordinated the expert groups. PHZ drafted the manuscript. Other experts participated in literature review, data collection, consensus discussion, and recommendations. All authors read and approved the final manuscript.

## Funding

This work was supported by the General Program of the National Natural Science Foundation of China (81772084) and the Shenzhen Medical Research Funds (B2402034).

## Competing interests

The authors declare that they have no conflict of interest.

## Data Availability

If reasonably requested, Dr. Pi-Hong Zhang can be contacted to obtain the original materials.

## Footnotes

Appendix A Supplementary data associated with this article can be found in the online version at doi:10.1016/j.mmr.2026.100058.
