# Supplementary material for: Chinese expert consensus on wound repair of venous leg ulcers (2025 edition)
Source: Mil Med Res. 2026 Jul 30;13(1):100058. doi: 10.1016/j.mmr.2026.100058 (PMC13452334; doi:10.1016/j.mmr.2026.100058)
Supplement: Supplementary file 1 [file mmc1.pdf]

List of Wound Repair Professional Committee of Chinese Medical Doctor Association

Consultants

Xiao-Bing Fu (The PLA General Hospital), Yong-Hua Sun (Beijing Jishuitan Hospital)

Members

Biao Cheng (People's Liberation Army General Hospital of Southern Theater Command), Hui Chen (Beijing Jishuitan Hospital), Guo-Xian Chen (The Second Affiliated Hospital of Zhejiang School of Medicine), Da-Hai Hu (The First Affiliated Hospital of Air Force Military Medical University), Jian-Hua Huang (Xiangya Hospital of Central South University), Yue-Sheng Huang (Institute of Wound Repair and Regeneration Medicine, Southern University of Science and Technology School of Medicine), Xiao-Yuan Huang (Xiangya Hospital of Central South University), Yu-Feng Jiang (The First Medical Center of General Hospital of PLA), Bing-Hui Li (Liyuan Hospital of Huazhong University of Science and Technology), Jing Li (The Second Affiliated Hospital of Air Force Military Medical University), Guang-Ping Liang (The First Affiliated Hospital of Army Military Medical University), Hong-Wei Liu (The First Affiliated Hospital of Jinan University), Shu-Hua Liu (The Third Hospital in Wuhan City), Xiao-Long Liu (People's Hospital of Xinjiang Autonomous Region), Yi-Wen Niu (Ruijin Hospital Affiliated to Shanghai Jiao Tong University School of Medicine), Yun-Chuan Pan (People's Hospital of Hainan Province), Yong-Tao Su (Shandong University of Traditional Chinese Medicine), Cong Wang (West China Hospital of Sichuan University), Yi-Bing Wang (Qianfo Mountain Hospital of Shandong Province), Bing Wen (Peking University People's Hospital), Xin-Yin Wu (Xiangya School of Public Health, Central South University), Wei-Guo Xie (The Third Hospital in Wuhan City), Hai-Lin Xu (People's Hospital of Peking University), Run-Gong Yang (The First Medical Center of General Hospital of PLA), Jian-Hua Zhan (The First Affiliated Hospital of Nanchang University), Yi Zhan (The Affiliated Hospital of Nantong University), Jia-Ping Zhang (The First Affiliated Hospital of Army Military Medical University), Pi-Hong Zhang (Xiangya Hospital of Central South University), Yi-Xin Zhang (Shanghai Ninth People's Hospital, Shanghai JiaoTong University School of Medicine), Tong-Bin Chu (The Second Affiliated Hospital of Dalian Medical University), Jun-Li Zhou (The Tenth Affiliated Hospital of Southern Medical University).

Table S1 Results of the Delphi questionnaire survey

Recommendations	Recommended (A)	Somewhat recommended (B)	Neutral (C)	Not recommended (D)	Somewhat not recommended (E)	A (%)
A comprehensive clinical assessment is essential for identifying the underlying etiology in patients with VLUs	30	1	0	0	0	96.8
Differential diagnosis is essential to distinguish VLUs from ulcers of other etiologies	27	4	0	0	0	87.1
The management of VLUs requires a multidisciplinary strategy that addresses both clinical manifestations and underlying etiology	29	2	0	0	0	93.5
Local compression therapy is beneficial for VLU healing and is considered a standard nursing intervention	27	4	0	0	0	87.1
In the absence of infectious complications, antibacterial agents are generally not indicated for VLU management	27	4	0	0	0	87.1
Prior to surgical interventions such as skin grafting, comprehensive and adequate WBP is essential	27	4	0	0	0	87.1
Radical debridement with a depth reaching the deep layer of the dense fibrous plate and extending beyond the liposclerotic region is more beneficial SG survival	26	5	0	0	0	83.9
For patients with a large ulcer area, a prolonged disease course, or suboptimal response to conservative treatment, WBP should be promptly initiated in combination with vascular intervention, followed by SG	27	4	0	0	0	87.1

Recommendations	Recommended (A)	Somewhat recommended (B)	Neutral (C)	Not recommended (D)	Somewhat not recommended (E)	A (%)
The sequence of this repair relative to the treatment of pathological veins should be determined based on the condition of the affected blood vessels, wound location and size, and presence of infection	26	5	0	0	0	83.9
Techniques such as the application of STSGs (e.g. meshed or microskin grafts, as well as the adoption of autologous epidermis and bilayer artificial skin grafts are effective therapeutic options for VLUs repair	25	6	0	0	0	80.6
The application of various tissue flap techniques has exhibited superior efficacy in repairing complex VLUs presenting with exposed bones, joints, or tendons, as well as recurrent and refractory conditions	24	5	2	0	0	77.4
Following VLU repair surgery, patients should be encouraged to enhance adherence to medical advice	29	2	0	0	0	93.5

Table S2 Levels of evidence

Level of evidence	Definition
A	Data derived from multiple randomized clinical trials or meta-analyses
B	Data derived from a single randomized clinical trial or large non-randomized studies
C	Consensus of expert’s opinion and/or small studies, retrospective studies, and registries

Table S3 Concentration, coordination, and authority of the consensus content (n=31)

Consensus	Mean	SD	Full score ratio (%)	Coefficient of variation	Kendall's coordination coefficient	Judgment coefficient (Ca, mean±SD)	Familiarity coefficient (Cs, mean±SD)	Authority coefficient (Cr, mean±SD)
Etiology and diagnosis					0.065 (P=0.157)	0.942±0.118	0.696±0.048	0.819±0.057
Etiology and clinical assessment	8.935	0.250	93.5	0.028				
Differential diagnosis	8.677	0.702	80.6	0.081				
Comprehensive treatment					0.065 (P=0.135)	0.923±0.112	0.683±0.093	0.803±0.072
Workflow and clinical pathway	8.839	0.523	90.3	0.059				
Compression therapy	8.968	0.180	96.7	0.020				
Systemic pharmacological therapy	8.645	0.877	83.8	0.101				
WBP and surgical debridement					0.031 (P=0.317)	0.929±0.122	0.683±0.075	0.806±0.073
WBP	8.355	1.018	67.7	0.122				
Surgical debridement	8.323	1.013	64.5	0.122				
Wound repair					0.011 (P=0.564)	0.942±0.092	0.655±0.109	0.799±0.101
Indications for skin-graft	8.581	0.807	77.4	0.101				
Timing for surgical intervention	8.774	0.560	83.8	0.064				
Wound repair strategy	8.710	0.588	77.4	0.068				
Recurrence prevention					0.034 (P=0.344)	0.929±0.110	0.683±0.077	0.806±0.059
Tissue flap transplantation	8.581	0.807	77.4	0.094				
POM and preventive strategies	8.903	0.301	90.3	0.034				

WBP. Wound bed preparation; POM. Postoperative management; SD. Standard deviation
